# Pilot Sleep Behavior across Time during Ultra-Long-Range Flights

**DOI:** 10.3390/clockssleep3040036

**Published:** 2021-09-23

**Authors:** Jaime K. Devine, Jake Choynowski, Caio R. Garcia, Audrey S. Simoes, Marina R. Guelere, Bruno de Godoy, Diego S. Silva, Philipe Pacheco, Steven R. Hursh

**Affiliations:** 1 Institutes for Behavior Resources, Inc., Baltimore, MD 21218, USA; jchoynowski@ibrinc.org (J.C.); shursh@ibrinc.org (S.R.H.); 2 Azul Linhas Aéreas Brasileiras, 06460-040 Sao Paulo, Brazil; caio.garcia@voeazul.com.br (C.R.G.); audrey.simoes@voeazul.com.br (A.S.S.); marina.guelere@voeazul.com.br (M.R.G.); bruno.godoy@voeazul.com.br (B.d.G.); silva.diego@voeazul.com.br (D.S.S.); philipe.pacheco@voeazul.com.br (P.P.); 3 Institutes for Behavior Resources, Johns Hopkins University School of Medicine, Baltimore, MD 21205, USA

**Keywords:** sleep score, sleep duration, sleep timing, time zones, COVID-19, aviation

## Abstract

Fatigue risk to the pilot has been a deterrent for conducting direct flights longer than 12 h under normal conditions, but such flights were a necessity during the COVID-19 pandemic. Twenty (N = 20) pilots flying across five humanitarian missions between Brazil and China wore a sleep-tracking device (the Zulu watch), which has been validated for the estimation of sleep timing (sleep onset and offset), duration, efficiency, and sleep score (wake, interrupted, light, or deep Sleep) throughout the mission period. Pilots also reported sleep timing, duration, and subjective quality of their in-flight rest periods using a sleep diary. To our knowledge, this is the first report of commercial pilot sleep behavior during ultra-long-range operations under COVID-19 pandemic conditions. Moreover, these analyses provide an estimate of sleep score during in-flight sleep, which has not been reported previously in the literature.

## 1. Introduction

Pilots operating routes that are long-haul (LH), defined as flight duty periods (FDPs) longer than 6 h, or ultra-long-range (ULR), defined as FDPs longer than 12 h, routinely suffer from fatigue due to sleep disruption [1,2,3]. Schedule mitigation and in-flight napping are fatigue countermeasures used to reduce sleep pressure during LH/ULR flights and aviation is one of the most regulated industries with regards to fatigue [4,5,6,7,8,9]. Maintaining a home base time zone schedule during ULR rosters may help pilots avoid fatigue related to circadian misalignment or jet lag [2]. However, local environmental and social time cues strongly influence even pilots who have been instructed to retain a home base time schedule [2].

Several studies have also shown that pilot sleep quality is diminished during lay-overs and in-flight rest periods [3,5,10]. Diminished sleep quality not only reduces total sleep duration, but is less restorative than sleep of equal duration in a bedroom environment [3]. The restorative value of sleep, as estimated through subjective fatigue and objective performance, is related to sleep architecture—namely, slow wave sleep (SWS) and rapid eye movement (REM) sleep stages [11,12]. Polysomnography (PSG) is the gold standard to assess sleep stages, but is considered an impractical method for collecting sleep information in operational environments [13]. Consumer sleep trackers cannot measure sleep architecture, but many offer a non-equivalent sleep score under the assumption that sleep stages N1 and N2 are comparable to light sleep, SWS is comparable to deep sleep, and REM is its own category [14,15,16,17]. It should be noted that while sleep score refers to sleep as being light or deep, CSTs do not measure the brain waves necessary to determine sleep depth [18,19]. Reliable estimation of sleep architecture during LH/ULR rosters, particularly during in-flight sleep, is important toward understanding the quality of sleep across aviation operations. Estimation of sleep scoring is a preliminary step toward that goal.

Pilot fatigue constitutes a well-anticipated threat to aviation safety, but the estimation of fatigue risk and sleep behavior for specific flight rosters has been informed by scientific examination of fatigue factors during actual or simulated aviation operations. Data cannot be collected during operations which never occur, but the COVID-19 pandemic resulted in unprecedented commercial aviation operations, including ULR direct round-trip flights between Brazil and China.

Operators adapted to the unique operating conditions imposed by global lockdowns as best as possible given the limited information and tools available to predict fatigue. Brazil-based Azul Airlines, for example, estimated pilot fatigue using a biomathematical model prior to conducting five separate humanitarian missions to China between May and July of 2020 [20]. During missions, pilots wore a validated wrist actigraph (the Zulu watch, Institutes for Behavior Resources Inc., Baltimore, MD, USA [21]) and reported the sleep duration and quality of their in-flight rest periods using a sleep diary. Each mission consisted of four flight legs between 11–15 h long each going from: (1) Brazil to a layover destination in Europe, (2) the layover destination to a destination in China to pick up COVID-19 relief supplies, (3) China to a return layover destination, and finally, (4) the return layover destination to the home airport in Brazil. Pilots were each provided a 9 h rest opportunity per FDP, and were instructed to remain on a home base schedule, i.e., west Brazilian local time (UTC-5).

The Zulu watch is a commercial sleep tracker designed for use in operational environments which has been validated against PSG and actigraphy for sleep-wake determination and against PSG for the estimation of sleep score [21]. Two-min epochs within sleep events which are recorded by the Zulu watch are categorized as either interrupted sleep, light sleep, or deep sleep. It should be noted that the Zulu watch estimates sleep score based on wrist movement using a tri-axial accelerometer and on-wrist detection using a galvanic sensor. Specific differences between NREM–REM sleep stages cannot be estimated by accelerometry alone, but wrist movement can identify bouts of immobility which are known to correspond to periods of restful sleep that could include NREM and REM [22,23]. Previous studies have compared sleep scoring in commercial wearables or mobile apps against PSG under the assumption that sleep stages N1 and N2 are comparable to light sleep, SWS is comparable to deep sleep, and REM is its own category [14,15,16,17]. The Zulu watch does not include a category for REM sleep.

The data analyzed in this manuscript was collected by Azul Airlines’ human factors team; secondary use of the data was granted to the science team at the Institutes for Behavior Resources (IBR). The goals of the current analyses are three-fold. The first goal is to describe observed sleep behavior during pandemic-specific ULR flight conditions and thus, establish an expectation of rest patterns in the hopefully unlikely event of future global public health emergencies. The second goal is to describe patterns of sleep behavior and sleep score across ULR in-flight and layover sleep events with respect to pilots’ flight schedules and local night as a precedence for future investigations. The third goal is to evaluate the accuracy of Zulu watch measures of sleep duration and sleep timing estimation in operations compared against sleep diary. The Zulu watch has been validated in the laboratory, but the true test of its utility is (a) the ability to accurately measure sleep timing and duration compared to self-report in real-world operations and (b) the ability of Zulu watch measurements to inform assumptions about sleep behavior in real-world situations such as ULR flight rosters. Agreement between Zulu watch measures of sleep timing (i.e., sleep onset time and offset time) and sleep duration, compared to sleep diary report of sleep timing and duration during FDP in-flight sleep, was examined using Pearson’s r correlation, paired samples t-tests, and Bland–Altman plots. Taking these three study goals together, this paper constitutes the first report of sleep behavior and estimation of sleep score during in-flight sleep for commercial pilots flying ULR missions across multiple time zones under global pandemic conditions.

## 2. Results

### 2.1. Pilot Participation

In total, 40 pilots flew the Brazil-China routes between May and July 2020 for Azul’s humanitarian missions. Missions ranged from 96 h to 132 h in length. Missions 1, 2, and 5 departed in the afternoon (between 14:00 and 16:00 UTC-5) while Missions 3 and 4 departed in the early morning (between 01:30 and 03:00 UTC-5). Each mission consisted of 4 FDPs which ranged in length from 11 to 14 h each, 1 turnaround period in China, which lasted between 3 to 6 h without deplaning, and 2 layover periods in Europe which lasted between 20 to 41 h. Thirty-two (32) out of 40 (80%) pilots crewing a COVID-19 humanitarian mission completed the sleep diary and 22 out of 40 (55%) wore a Zulu watch continuously during the mission period. Twenty (20; 50%) pilots completed both the sleep diary and wore the Zulu watch. Only pilots who both completed the sleep diary and provided Zulu watch data (N = 20) have been included in these analyses. Fifteen (N = 15) participants provided Zulu and diary data for all 4 flight legs; N =3 participants provided Zulu and sleep diary data for 3 out of the 4 flight legs; N =1 participant provided Zulu watch data for all FDPs, but only completed the sleep diary for 3 out of 4 FDPs and N = 1 participant completed the sleep diary for all 4 FDPs, but only wore the Zulu watch for 3 out of the 4 flight legs.

### 2.2. Sleep Timing and Sleep Duration across Mission Hours

**Figure 1:**
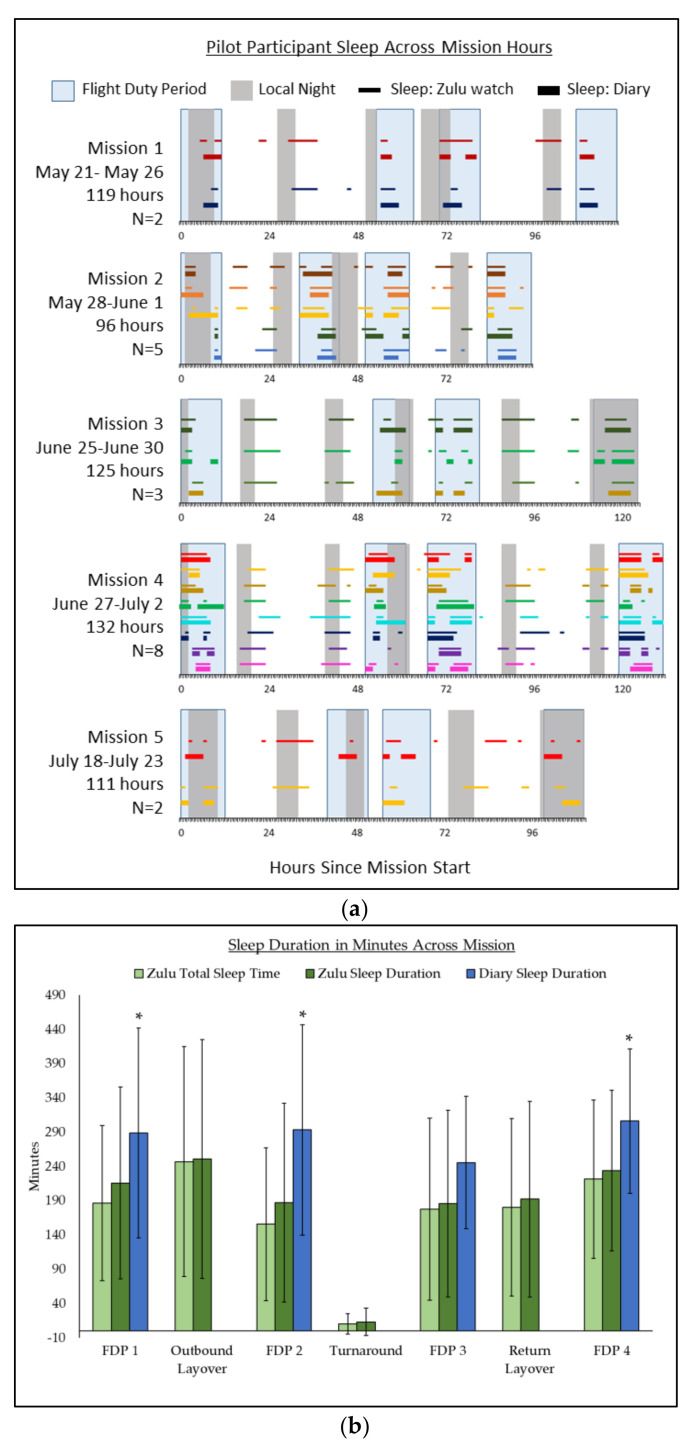
Pilot Participant Sleep During COVID-19 Humanitarian Missions. Sleep timing and sleep duration across COVID-19 humanitarian missions as measured by Zulu watch and sleep diary. (**a**) Pilots’ sleep episodes occurring across each mission hour as measured by Zulu watch compared to sleep diary reports of sleep during FDPs are depicted by color-matched thin (Zulu watch) and thick (diary) lines. FDPs are indicated by light blue boxes; local night is indicated by gray boxes. (**b**) Comparison of sleep duration as measured by Zulu watch and Zulu watch TST compared to diary sleep duration across all mission FDPs, layovers, and turnaround. * represents significance at *p* ≤ 0.05.

Pilots reported between 0 to 3 separate sleep events per FDP by diary. In comparison, Zulu watches recorded between 0 to 6 sleep events for the same FDP. FDPs include time allocated for commuting and preparation. Sleep duration was not reported during layover or turnaround. There was only one instance in which a pilot did not report any sleep and no sleep event was recorded by the Zulu watch during a flight leg. For FDPs during which sleep occurred, sleep duration ranged between 30–520 min as reported by diary and 20–518 min for Zulu watch. Sleep occurred across 24 h of the day. On average, pilots reported sleeping for 237 ± 43 min and sleeping once within a 24–h period. Average sleep duration per 24 h as measured by the Zulu watch was 368 ± 55 min; average TST per 24 h was 321 ± 56 min as measured by the Zulu watch, with pilots sleeping between 2 to 3 times within a 24-h period in contrast to sleep diary report of 1 sleep episode. Figure 1 depicts each pilot participants’ sleep behavior with respect to mission FDPs and local night across all hours of the missions. Time is reported in hours elapsed since mission start rather than in base, local, or GMT time to avoid confusion about sleep behavior as pilots circumnavigated the globe.

Pilot sleep opportunities during FDPs were determined in-flight by the crew. Sleep opportunities were decided *ad libitum* by pilots during layovers, and pilots were instructed not to sleep during the turnaround periods in China. Pilots were confined to the aircraft during turnaround in China, but their activities were not restricted during layover periods. The timing of pilot sleep with respect to the end of the previous FDP or the start of the subsequent FDP ranged from 0 min to 2527 min (approximately 42 h). In contrast, pilot sleep began, on average, 50 ± 70 min after the onset of local night. There were no differences between sleep onset with regard to local night depending on whether pilots were sleeping in-flight or during a layover period (t = 0.19, *p* = 0.85).

### 2.3. Sleep Quality and Sleep Score across Missions

**Figure 2:**
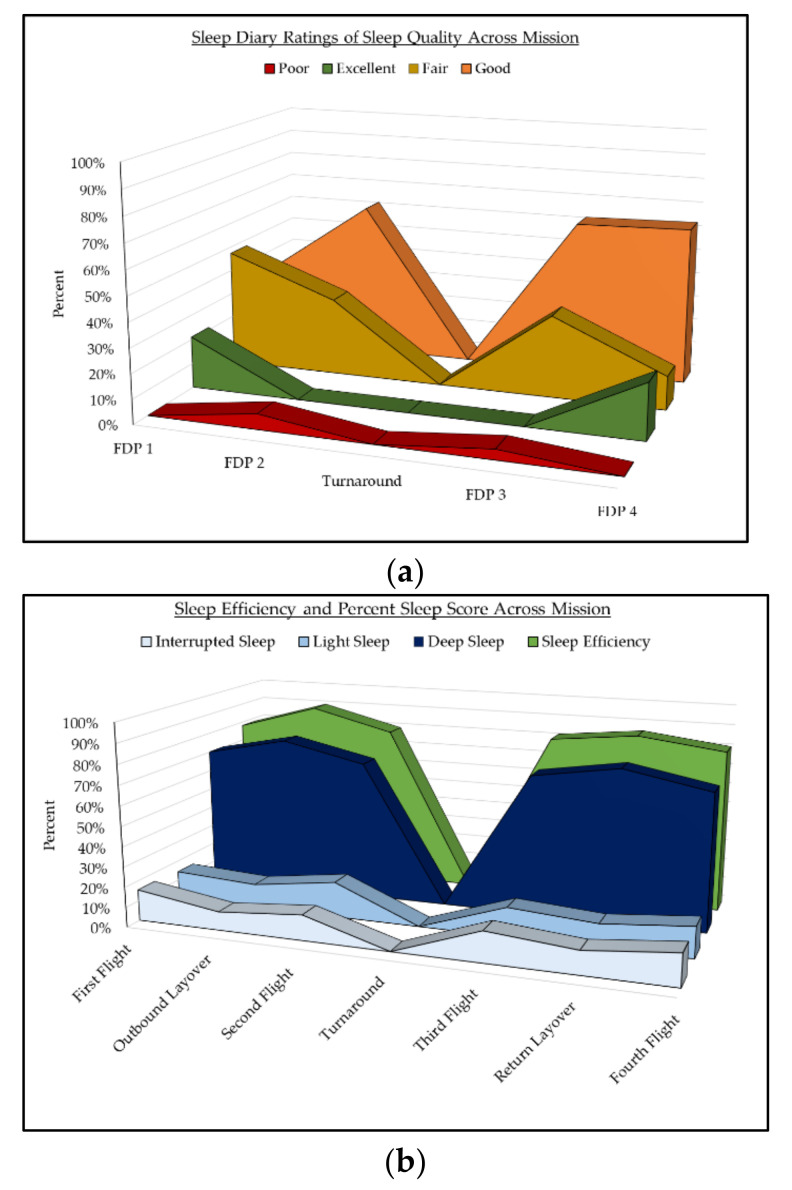
Subjective Sleep Quality, Sleep Efficiency, and Sleep Score Across Mission Flight Legs. (**a**) Diary reports of sleep quality during in-flight sleep across all missions FDPs. Pilots only reported sleep quality during in-flight sleep. The graph depicts “Excellent” sleep in green, “Good” sleep in orange, “Fair” sleep in yellow, and “Poor” sleep in red. The breakdown of sleep quality ratings by percentage are reported for each flight leg. (**b**) Sleep efficiency and sleep score categories as measured by Zulu watch.

Distribution of sleep quality across categories (Excellent, Good, Fair, and Poor) is depicted in Figure 2a. Pilots reported “Good” sleep quality for the majority of in-flight sleep. There were 81 diary reports of sleep quality in total. Percentages of interrupted, light, deep sleep, and overall sleep efficiency (SE) as measured by Zulu watch are depicted in Figure 2b. Average SE during in-flight sleep was 85% ± 8%. Pilots’ sleep was classified as deep sleep for 70% ± 12%, light sleep for 15% ± 7%, and interrupted sleep for 15%±8% of TST. Differences in sleep quality ratings and Zulu watch sleep score percentages were statistically non-significant across all FDPs and missions (all *p* > 0.05).

### 2.4. Agreement between Zulu Watch and Diary Measurements of Sleep

**Figure 3:**
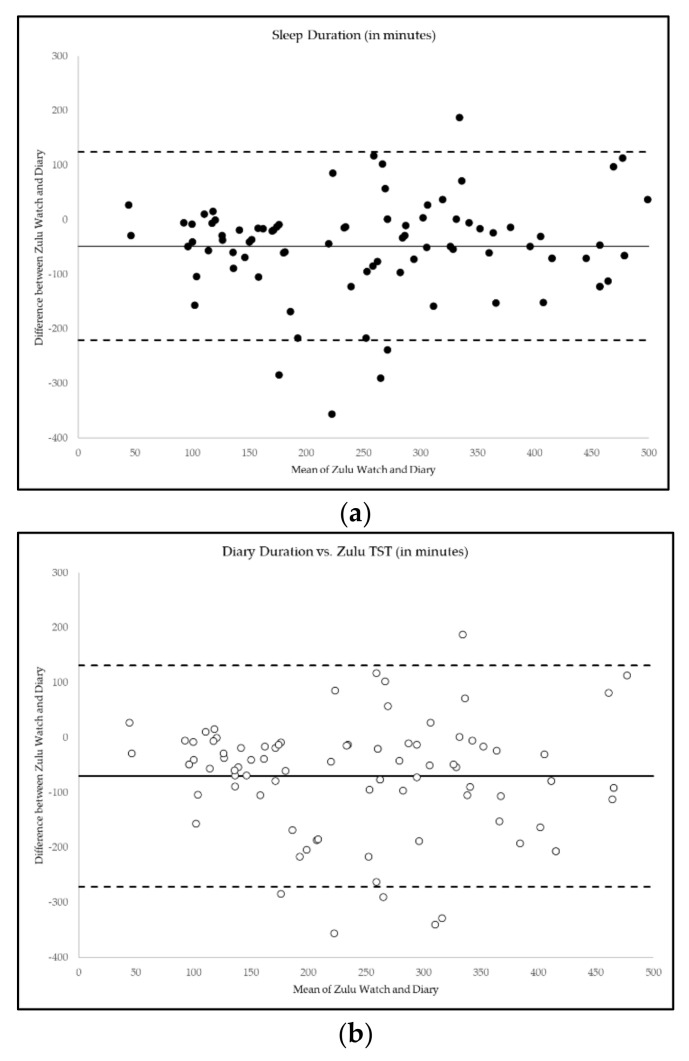

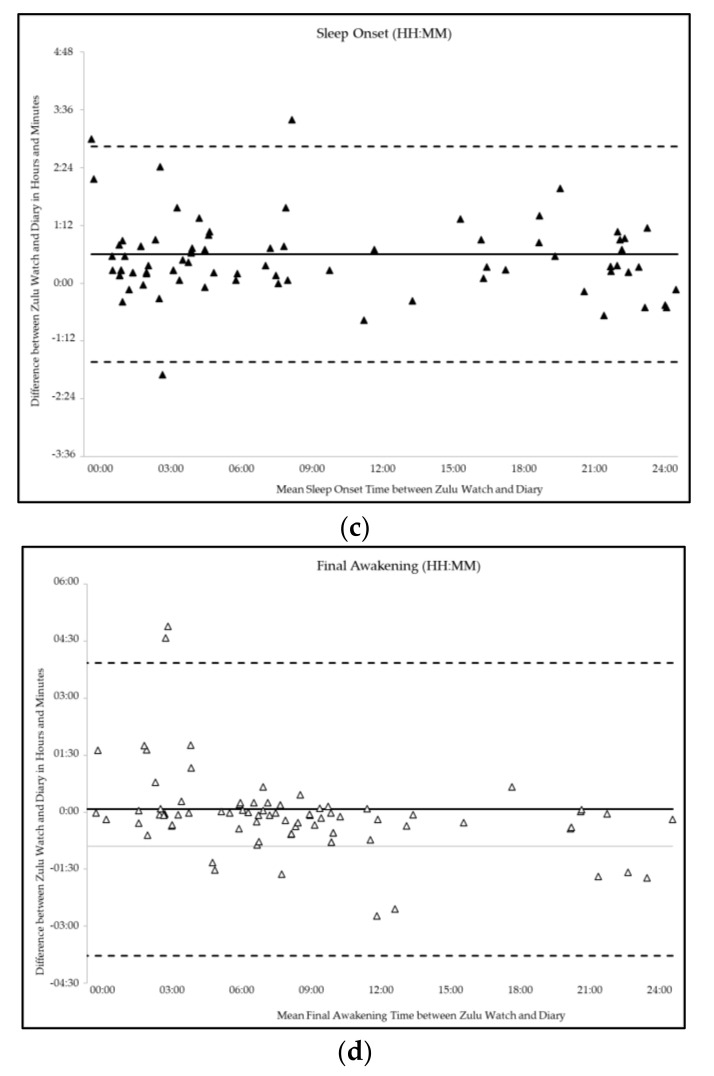
Agreement between Zulu Watch and Diary Measures of Sleep Timing and Duration. (**a**) Bland-Altman plots of the differences (*y*-axis) between the Zulu watch and diary measures of sleep versus the mean of the two measurements (*x*-axis). Bias is represented by the solid line (―). Upper and lower limits of agreement (LOAs) are represented by the dashed lines (- -). Narrower LOAs indicate relatively less variability between measures. Agreement is shown for (**a**) Zulu watch and diary measures of sleep duration (●); (**b**) Zulu watch TST and diary sleep duration (◦) (**c**) time of sleep onset (▲); (**d**) time of final awakening (Δ).

Average sleep duration per sleep event was 281 ± 126 min by sleep diary compared to 204 ± 134 min for Zulu watches. Diary sleep duration was positively correlated with Zulu sleep duration (r = 0.75, *p* ≤ 0.001) and TST (r = 0.75, *p* ≤ 0.001), but paired samples t-tests showed that diary reports of sleep duration were significantly higher than Zulu watch sleep duration (t = 5.24, *p* ≤0.001) or TST (t = 6.49, *p* ≤ 0.001). Sleep onset times were positively correlated between Zulu watch and diary (r = 0.74, *p* ≤ 0.001), as was time of final awakening (r = 0.62, *p* ≤ 0.001). Between 40%–56% of the variance in Zulu sleep duration (R^2^ = 58%) or TST (R^2^ = 41%) could be explained by sleep diary measurements. Diary-reported sleep onset explained 55% of variance in Zulu watch sleep onset (R^2^ = 58%) and diary-reported final awakening explained 38% of Zulu watch final awakening (R^2^ = 45%). Sleep onset time and time of final awakening were not significantly different between Zulu watch and diary (all *p* > 0.05). Figure 3 summarizes the Bland–Altman plots of the mean difference, bias, and limits of agreement for Zulu watch and sleep diary measures of sleep duration, diary sleep duration compared to Zulu TST, time of sleep onset, and time of final awakening.

## 3. Discussion

It must first be mentioned that the circumstances of the 5 ULR flights profiled in this manuscript are exceptional. The purpose of Azul’s humanitarian missions was to bring respirators, COVID rapid tests, and medical supplies from mainland China back to Brazil. The humanitarian goal of these missions served as a uniquely motivating factor for each pilot who participated in the missions. Azul Airlines had not previously conducted flights to China, and the pilots were unfamiliar with the destination airports within China. While the missions were conducted by commercial airline pilots on a commercial aircraft, there were no passengers or cabin crew aboard. Pilots were permitted to sleep in either crew rest facilities or in the business class section, per their preference. Moreover, pilots were restricted from leaving the aircraft while in China, and while they were permitted to move freely during layovers in Europe, shutdowns related to COVID-19 most likely limited the availability of social activities. Because these ULR flights were conducted under unprecedented global pandemic conditions, pilot behavior may not generalize to all ULR operations.

Pilots slept between 5 to 6 h per 24-h period despite being afforded 9 h of sleep opportunity per FDP and between 20 to 40 h of free time during layovers. Using sleep diary data, sleep duration was less than 4 h per 24-h period. It must be noted that sleep diary did not include reports of sleep during layover periods and should not be considered an accurate total of 24-h sleep. Estimations of daily sleep duration were further limited due to the fact that flights departed Brazil at variable hours of the day and crossed multiple time zones continuously throughout the mission. Parameters for “daily” estimations of sleep are normally defined with hourly cutoffs, such as noon to noon [24]. These cutoffs could have been retained using Brazil time (UTC-5) except that 3 of the 5 missions departed in the afternoon while 2 departed shortly after midnight. To avoid complications when comparing between all flights, “daily” estimates of sleep were defined as sleep intervals ending within a 24-h period starting with mission hour 0. Sleep duration per 24-h period were not significantly different depending on whether flights departed during the afternoon versus the early morning. While preliminary, these data suggest that pilots during ULR travel may experience shortened sleep (~5–6 h per day) even when provided sufficient opportunities for sleep and under conditions of limited social distractions. Insufficient pilot sleep during ULR flights could have implications for fatigue risk management or pilot well-being.

Despite being instructed to remain on home base time, the logistical necessity of coordinating in-flight sleep opportunities with co-pilots and severe limitations to social time cues, pilots tended to initiate sleep within an hour of the onset of local night. The clustering of pilot sleep around local night can most clearly be seen in Figure 1a. The timing of pilot sleep with respect to FDPs was vastly more variable, occurring anywhere from 0 to 42 h apart. These findings indicate that environmental light cues may influence sleep behavior over the course of ULR transmeridian travel over and above logistical considerations such as the timing of work or adherence to a home schedule.

Understanding the quality of pilot sleep during ULR operations is important for the mitigation of fatigue. There is a lack of previous data examining sleep quality in the context of aviation or in-flight sleep. In this study, neither subjective nor objective sleep quality changed significantly over the course of the mission. Sleep efficiency remained in a normal range (above 80%) throughout all FDPs and layovers, and pilots largely rated their sleep as “good” or “fair”. Estimation of sleep score remained consistent as well, with the majority of TST being spent in “deep sleep”. However, actigraphy devices have a problem with low specificity [25,26,27], meaning that they are not very good at picking up awakenings during sleep intervals. The specificity of the Zulu watch to identify awakenings during a sleep interval compared to PSG under laboratory conditions is 26% [21]. Moreover, while sleep score estimation by the Zulu watch has been tested against gold-standard PSG under laboratory conditions, no investigations of sleep score or sleep architecture have ever been conducted during in-flight sleep. While these data represent a step towards understanding the impact of ULR travel on sleep quality, the limitations of the technology must be acknowledged. Extensive future research and advancements in device specificity will be required in order to determine whether sleep quality is truly resilient to transmeridian travel or not.

Another aim of the current analyses was to evaluate agreement between sleep diary and Zulu watch measures of sleep timing and duration. Zulu watch measures of sleep were moderately, though not strongly, correlated with diary measures. Time of sleep onset and final awakening were similar between diary and Zulu watches. However, pilots consistently reported longer sleep duration than was recorded by the Zulu watch. This finding is consistent with previous findings that sleep duration measured by actigraphy is shorter than sleep diary report [28,29,30]. Pilots only reported in-flight sleep, so we could not test agreement between diary and the Zulu watch before or after the mission or during layover periods. It is possible that turbulence or background movement of the airplane in flight could falsely register an awakening on the Zulu watch. However, considering the low specificity of the Zulu watch and actigraphy devices in general, this possibility is not highly likely.

In some ways, the testing of Zulu watch measures of sleep against a diary was akin to comparing apples and oranges. The Zulu watch considered periods of awakening as the termination of a sleep episode, while pilots may have reported the total amount of time during which they attempted sleep, regardless of whether any sleep occurred. For this reason, multiple Zulu watch sleep intervals occurred over the course of one diary entry. Despite our best efforts to objectively compare the two measures, researcher bias may have influenced the results. An additional limitation is that the data were collected for operational purposes rather than for scientific study. Since the available data was restricted to the mission period, this precluded the examination of the role of pilot demographics, sleep or medical history, or other potential intervening variables on rest patterns during the missions. The Human Factors team at Azul did an exceptional job of collecting quality data which could be evaluated for scientific purposes *post hoc*, but this was not a controlled experimental study.

Validation testing methodology has been established for a laboratory environment [31], but there is little guidance for what constitutes proper validation of sleep measurement in a real-world environment. Validity testing against sleep diary is one method that is feasible in an operational environment, but relies on the assumption that diaries are accurate measures of sleep. Diary report of sleep can differ from gold-standard PSG or actigraphy measurements [28,29,32,33]. This constitutes a limitation to sleep research in general, and closing the validation gap is a goal for future research [34]. Previous studies have compared self-report to actigraphy but these comparisons have been made either under controlled conditions or in specialty populations that are not directly comparable to airline pilots [24,28,29,30,32,35]. Explained variance between measures of sleep duration or timing have been reported in these previous studies. In this study, explained variance for comparisons of sleep timing and duration was below or slightly above 50%. It is difficult to interpret this finding without context from the literature, which constitutes a study limitation. Understanding sleep patterns during duty periods is an important step forward toward mitigating fatigue in operational environments, but the distraction caused by data collection during a mission is itself a safety risk. While it is impossible to say whether the subjective assessment of sleep or the objective measurement is more representative of actual sleep under the circumstances of testing in the field, it is worthwhile to note that pilots were able to passively provide data through the Zulu watch more consistently over the mission than they were able to provide sleep diary report.

## 4. Materials and Methods

### 4.1. Participants

Participants were recruited through Azul Airlines’ Human Factors Safety Department. Participants provided written informed consent for their participation. All missions were considered eligible for participation regardless of gender, ethnicity, age (over 18), sleep habits, or health status. Secondary use of de-identified data for research purposes was approved by Salus Institutional Review Board and these analyses were conducted in accordance with the Declaration of Helsinki.

### 4.2. Procedures

Mission flights were designed to be carried out with 2 relay crews consisting of 8 pilots. There were 4 flight legs to each mission: (1) Brazil to a European layover destination; (2) layover to China; (3) China to a return layover destination; and (4) layover to Brazil. Each flight leg was approximately 12 h and the planned available rest time for each crew member per stage was approximately 9 h. The crews were organized so that all pilots would be available to work during any flight leg and that no one pilot would need to fly extra time. In-flight rest periods were freely chosen by the crew during the mission. Each flight leg was approximately 12 h and the available rest time for each crew member per stage was approximately 9 h. Aircrew were instructed to remain on home base Brazilian time throughout the mission.

Pilots were assigned the Zulu watch (Institutes for Behavior Resources, Inc., Baltimore, MD, USA [21]) in May 2020 prior to COVID-19 support missions and wore the watches continuously until the completion of their mission (between May and July, 2020). Crews returned the watch to airline researchers directly upon returning to Brazil from their mission. Data were downloaded by airline researchers using the Zulu Data Extraction application (Institutes for Behavior Resources, Inc., Baltimore, MD, USA, Version 2.0). Pilots completed a sleep diary during FDPs.

### 4.3. Sleep Measures

#### 4.3.1. Zulu Watch

The Zulu watch hardware device collects activity data in 2-min epochs and automatically scores sleep duration and sleep efficiency on-wrist based on a proprietary algorithm for sleep–wake determination. Devices were programmed to detect multiple sleep intervals per day and can detect sleep intervals which are as short as 20 min in duration. Data were then exported as one summary file of all scored sleep interval information and as multiple 2-min epoch-by-epoch (EBE) data files for each day during the mission study period. Zulu watch scored sleep interval summary files included sleep onset time and sleep offset time reported as mm/dd/yyyy hh:mm, sleep duration in minutes, and SE as a percentage for any events determined to be a sleep interval by the Zulu watch.

Epoch data are scored as on-wrist “On” or off-wrist “Off”. Epochs are scored in a separate data column as 0 for periods of wake, 1 for restless or interrupted sleep, 2 for light sleep, and 3 for deep sleep. The Zulu watch uses a proprietary algorithm to estimate sleep score using only motion and on-wrist detection and cannot differentiate between sleep stages. Zulu watch sleep scoring should be considered an estimation of locomotor inactivity rather than an estimate of neurophysiological sleep architecture.

#### 4.3.2. Sleep Diary

The pilots reported the start time and end time (as mm/dd/yyyy hh:mm), sleep duration in hours and minutes, and categorical subjective quality of any sleep intervals occurring during FDPs. Subjective sleep quality was rated on a 4-point scale as either Poor, Fair, Good, or Excellent by pilots. Pilots were not asked to complete the sleep diary during layovers or ground time in China. All times were reported in Brazilian time.

### 4.4. Reformatting Data for Consistency between Zulu Watch and Diary Measurements

The Zulu watch automatically determines sleep onset and sleep offset regardless of whether the wearer is still attempting sleep. For this reason, while the Zulu watch can provide a measure of sleep duration similar to total sleep time (TST), it does not provide an estimate of time in bed (TIB). Conversely, pilots reported the amount of time that they dedicated to sleep, which more closely resembles a measurement of TIB. However, the term “time in bed; TIB” cannot be considered an accurate description of sleep opportunities in the current analyses since none of the sleep events reported in this manuscript occurred in a bed or bedroom environment. Because of the constitutional difference in data reporting, multiple Zulu watch sleep events occurred over the course of a single diary-reported event. In order to most accurately compare Zulu watch measurements against diary, all minutes of sleep duration recorded by Zulu watch within proximity to 1 diary-reported sleep event were summed. Sleep onset time and sleep offset time were selected from the earliest occurring Zulu watch sleep interval and last occurring Zulu watch sleep interval data, respectively. An estimate of time dedicated to sleep as measured by the Zulu watch was computed as the minutes occurring between the earliest-occurring Zulu watch sleep onset time and the last-occurring sleep offset time for comparison against diary sleep duration. For the purposes of these analyses, sleep duration will refer to time dedicated to sleep (a proxy for TIB), and TST will refer to the time recorded as sleep by the Zulu watch in minutes.

### 4.5. Data Analysis

All statistics were computed using Excel 2013, STATA version 15, and RStudio version 1.3.959. Sleep duration is defined as time (in minutes) dedicated to sleeping based on Zulu watch or diary. Total sleep duration per flight leg was computed by summing all minutes of sleep recorded or reported occurring during each flight leg. Total sleep duration across 24 h were computed by summing all minutes of sleep recorded or reported in which the wake-up time occurred within a 24-h period starting at mission hour 0. The number of daily sleep intervals (DSI) was determined by counting the number of sleep events recorded or reported for each 24-h period starting at mission hour 0. All date/time data were converted to west Brazilian home base time zone (UTC-5) for consistency. Sleep onset and offset times as reported by Zulu and sleep diary were converted from UTC-5 date time format to numeric values for statistical analysis. Distance between sleep onset and FDPs or local night were computed by subtracting the sleep start time from the end time of previous FDP or start time of subsequent FDPs or by subtracting sleep start time from the start time of local night. Local night start times were extracted from the Sleep, Activity, Fatigue, and Task Effectiveness Fatigue Avoidance Scheduling Tool (SAFTE-FAST) biomathematical modeling software. Differences between sleep distance from night by FDP versus layover were examined using Student’s t-test. Differences in sleep quality ratings and Zulu watch sleep score percentages across flight legs and between missions were compared using repeated measures mixed model analysis. Percent explained variance was evaluated using adjusted R^2^ values. Paired samples t-tests were run to compare differences between Zulu watch and diary-reported measures of in-flight sleep. Mean difference scores were additionally computed between sleep onset time, sleep offset time and sleep duration. Bland–Altman plots examined the mean difference between measures of sleep and single sample t-tests were conducted to determine if a statistically significant difference existed between mean difference scores. Limits of agreement were computed (mean difference ± 1.96 SD) to indicate the range in which the differences between the two measures would occur with 95% probability [36]. The strength of the association between Zulu watch and diary report for measures of sleep onset, offset, and duration was calculated using Pearson correlation coefficients.

## 5. Conclusions

This is the first report of sleep behavior and sleep score estimation in pilots operating ULR flights during global pandemic conditions to our knowledge. Pilots tended to sleep during local night despite being instructed to adhere to home base time schedule and having to coordinate sleep opportunities with their co-pilots. Subjective sleep quality, SE, and percentage of interrupted, light, and deep sleep remained consistent across the missions, and were not indicative of diminished sleep quality. Zulu watch and diary measures of sleep were similar, but pilots reported longer sleep duration than was measured by the Zulu watch. These analyses can help inform the management of fatigue risk in the planning or future ULR flights or pandemic flight conditions.

## Data Availability

Data for this study is owned by Azul Airlines and is not publicly available.
